# Clinical predictors of pseudoprogression in glioblastoma: a retrospective cohort analysis

**DOI:** 10.1007/s11060-025-05299-0

**Published:** 2025-10-24

**Authors:** L. J. N. Hoosemans, L. F. Panayotopoulos, S. M. J. van Kuijk, M. Verduin, M. H. M. E. Anten, R. C. O. S. Pasmans, J. M. Schellekens, S. M. H. Huijs, S. R. Verhoeff, F. W. P. J. van den Berkmortel, L. C. Steggink, L. Ackermans, O. E. M. G. Schijns, O. P. M. Teernstra, K. Hovinga, J. C. Beckervordersandforth, I. Compter, D. B. P. Eekers, A. A. Postma, M. A. Vooijs, M. P. G. Broen, A. Hoeben

**Affiliations:** 1 Department of Radiation Oncology (MAASTRO), GROW School for Oncology and Reproduction, Maastricht University Medical Center+, Maastricht, Netherlands; 2 Department of Medical Oncology, GROW School for Oncology and Reproduction, Maastricht University Medical Center+, Maastricht, Netherlands; 3 Department of Clinical Epidemiology & Medical Technology Assessment, Maastricht University Medical Center+, Maastricht, The Netherlands; 4 Department of Neurology, GROW School for Oncology and Reproduction, Maastricht University Medical Center+, Maastricht, The Netherlands; 5 Department of Neurology, Zuyderland Medical Center, Heerlen, The Netherlands; 6 Department of Medical Oncology, Zuyderland Medical Center, Sittard-Geleen, The Netherlands; 7 Department of Neurosurgery, Maastricht University Medical Center+, Maastricht, The Netherlands; 8 Academic Centre for Epileptology, Maastricht University Medical Center+ and Kempenhaeghe, Maastricht, Heeze, The Netherlands; 9 Department of Pathology, Maastricht University Medical Center+, Maastricht, The Netherlands; 10 Department of Radiology and Nuclear Medicine, Maastricht University Medical Center+, Maastricht, The Netherlands; 11 Mental Health and Sciences Research Institute (Mhens), Maastricht University, Maastricht, The Netherlands

**Keywords:** Glioblastoma, Pseudoprogression, Chemoradiotherapy, Predictive factors, MGMT

## Abstract

**Purpose:**

Distinguishing pseudoprogression (PsP) from true progression (TP) in glioblastoma (GBM) remains a diagnostic challenge, yet is essential for guiding treatment and counseling prognosis. This study retrospectively assessed the incidence, clinical predictors, and survival impact of PsP compared to TP.

**Methods:**

Patients with surgically treated GBM and postoperative (chemo)radiotherapy in two Dutch hospitals (2006–2021) were included. Reports of magnetic resonance imaging (MRI) scans performed 4 months post-radiotherapy and at 3-month intervals, as well as reports of MRI scans prompted by neurological decline, were evaluated for PsP, TP, or mixed response (MR). Associations with clinical, tumor, and treatment characteristics and overall survival (OS) were analyzed.

**Results:**

Of 424 GBM patients, 175 were eligible for PsP analysis. The incidence of PsP was 29.1%, and PsP was associated with longer OS (median 16.6 months, 95% CI 12.0–21.2) compared to MR (14.1 months, 95% CI 11.1–17.2) and TP (11.6 months, 95% CI 10.0–13.2; *p* = 0.010). However, PsP occurring < 4 months after chemoradiotherapy was linked to shorter OS (11.3 months) than PsP > 4 months (17.4 months; *p* = 0.027). Male sex was significantly associated with outcome in univariate analysis, showing a trend toward significance in multivariate analysis. Treatment completion remained significant only in the multivariate model.

**Conclusion:**

PsP is associated with improved survival compared to TP, though early-onset PsP portends poorer outcomes. None of the evaluated factors were a significant predictor of PsP in both univariate and multivariate analyses. Future research should focus on validating molecular markers, and refining PsP definitions using standardized criteria.

**Supplementary Information:**

The online version contains supplementary material available at 10.1007/s11060-025-05299-0.

## Introduction

Glioblastoma (GBM), isocitrate dehydrogenases (IDH) wild type, is the most common and aggressive primary brain tumor [1]. Despite optimal treatment of maximally safe resection and concomitant chemoradiation with adjuvant temozolomide, median OS remains a mere 15 months [2]. The standard treatment protocol, as developed from the EORTC 26,981/22,981 study (from now on named “Stupp protocol”), recommended for patients below the age of 65 years with a favorable performance status, involves radiotherapy delivered at a total dose of 60 Gy in 30 fractions over 6 weeks, administered concurrently with temozolomide, followed by six cycles of adjuvant temozolomide [2]. For elderly (>70 years) or frail patients, several studies have been performed with adjusted protocols to reduce treatment burden while maintaining efficacy [3–6]. Of these, the treatment protocol according to the EORTC-26062-22061 study (from now on named “elderly protocol”) is used in our clinical centers. This protocol involves hypofractionated radiotherapy, consisting of 40 Gy in 15 fractions, concurrent with temozolomide, also followed by adjuvant temozolomide [3]. 

Since the introduction of these treatment schedules, where temozolomide is given concurrently with radiotherapy to act as a radiosensitizer, an increase in the rate of pseudoprogression (PsP) has been observed [7]. PsP occurs in 20–30% of GBM patients following chemoradiation [8–12]. PsP is defined by the Response Assessment in Neuro-Oncology (RANO) criteria as an increase in contrast enhancement on magnetic resonance imaging (MRI) following radiotherapy, mimicking tumor progression but eventually improving without a change in therapy [13–15]. This contrast enhancement is most likely attributable to a pronounced local tissue response involving inflammation and increased permeability of the blood-brain barrier (BBB) [16]. PsP predominantly occurs within the first 6 months post-chemoradiation. Radiation necrosis is a severe and more permanent local tissue reaction to radiotherapy with similar features to PsP and is challenging to differentiate. It typically develops 3–12 months after therapy or even years later and becomes more likely than PsP beyond the 6-month period [14, 17]. Early differentiation between PsP and TP is essential to enable timely adjustment of therapy in cases of TP. Additionally, PsP is associated with an improved OS rate [10, 15, 18–20].

Currently, MRI contrast enhancement follow-up is the gold standard for tumor evaluation, although different additional imaging techniques are used to increase diagnostic accuracy, including MRI with perfusion weighted imaging (PWI) [17, 21, 22]. Clinical predictors of PsP, such as demographic features, resection type and treatment scheme may also aid in clinical decision making. To this end, several factors potentially associated with PsP have been studied. O^6^-methylguanine-DNA methyltransferase (MGMT) promoter methylation is the most extensively studied molecular marker and has been associated with an increased likelihood of PsP [8, 10, 23–26]. Nevertheless, not all studies have demonstrated a correlation between MGMT promoter methylation and the development of PsP [27, 28]. Some studies analyzing potential causality between oncogenic drivers and PsP have been constrained to a limited number of variants with small sample sizes [18, 27, 29] and others have yielded inconclusive or statistically insignificant results [9, 25]. 

The primary aim of our study was to explore a range of clinical and molecular characteristics predictive of PsP in glioblastoma patients. Knowledge of these factors can potentially aid in clinical decision making and counseling the patients in challenging cases. Our secondary aim was to evaluate OS with respect to the occurrence of PsP and correlation to clinical features.

## Methods

### Patient cohort

In this retrospective cohort study patients diagnosed with GBM between January 1st, 2006, and December 31st, 2021, at either Maastricht University Medical Center+ (MUMC+; NL) or Zuyderland Medical Center (ZMC; NL) were included. Eligible participants were 18 years of age or older at the time of diagnosis and had a histologically and molecular confirmed GBM. Patients with IDH mutant tumors were excluded to match the 2021 WHO Classification of Tumors of the Central Nervous System [30]. Patients with missing follow-up MRI scans were also excluded, as accurate definitions of PsP and TP could not be defined. Patients who received chemotherapy monotherapy were excluded, as radiation is typically required for the development of PsP.

### Data collection

Patient data were retrieved from electronic patient files. The collected data included demographic information, tumor characteristics, treatment details, and outcomes. Treatment details include first line of treatment, differentiating between the standard Stupp protocol [2] and the shorter course elderly chemoradiation protocol. As the elderly protocol has only been published and implemented in 2017 [3], this treatment cohort has a smaller sample size. Patients treated before 2017 received either Stupp or radiotherapy monotherapy.

For PsP assessment, reports of MRI scans made by neuro-radiologists were used. MRI scans at 4, 7, 10 and 13 months post-chemoradiation were utilized, as well as additional MRIs performed because of neurological deterioration. MRI assessments comprised pre-gadolinium T1-weighted, post-gadolinium T1-weighted, T2-weighted, and fluid-attenuated inversion recovery (FLAIR) images. Scoring was based on contrast enhancement, increased perfusion (when performed) and change in treatment (as decided in the multidisciplinary neuro-oncology team). Perfusion ratios (maximum relative cerebral blood volume, rCBV), above 3 were generally accepted as increased perfusion, based on the ratio range of 1.49–3.10 in previous studies [31, 32]. Pathological confirmation of PsP was not available in any of the patients, as this is not part of the national guideline for glioma treatment and follow-up in the Netherlands.

In GBMs diagnosed before 2014, MGMT promoter methylation was analyzed using multiplex ligation-dependent probe amplification (MLPA), with a methylation threshold of 25%. After 2014, methylation-specific PCR (MSP) was used to define MGMT promoter methylation, with a methylation threshold of minimum 15%. The Illumina TruSight Oncology 500 (TSO500) next generation targeted sequencing panel was applied to evaluate other genetic alterations at both centers. Variants of unknown significance (VUS) were excluded from analyses.

### Outcomes

Three distinct outcomes have been established to distinguish PsP from TP, as based on a combination of the RANO criteria, as well as more recent additional techniques applied. This includes the evaluation of increased contrast enhancement with evaluation of a consequent scan within 3 months, as described by the RANO [13]. Additionally, we used perfusion MRI scans (assessing relative cerebral blood volume, rCBV) to further differentiate PsP from TP, as based on numerous additional studies [14, 22, 33]. This classification also reflects current clinical practice. PsP was defined as an increase in contrast enhancement without increased perfusion (normal relative cerebral blood volume, rCBV) on MRI (as assessed by neuro-radiologists in our centers), and/ or with a report of no change in treatment and without progression on MRI scan within 3 months after the first scan. TP was defined as an increase in MRI contrast and perfusion (elevated rCBV, when MRI perfusion was performed), and/ or with a change in treatment. If patients first demonstrated increased contrast enhancement without an increased perfusion and/or no change in treatment (suggestive of PsP), but TP occurred on a subsequent MRI within three months follow-up, this was defined as a mixed response (MR). For comparative analyses, patients were categorized based on the first event that occurred (PsP, TP or MR) during follow-up, starting from the first MRI evaluation after completing the concurrent chemoradiation regimen.

OS was defined as the duration from the date of primary surgery (diagnosis date) to the date of death from any cause. For patients last known to be alive, OS data were censored at last follow-up visit. Follow-up data were collected until December 31, 2021.

Early and late PsP were distinguished by using 4 months (from end of chemoradiation) as the cut-off time point. This cut-off was chosen as to include completion of concurrent chemoradiation as well as the first follow-up MRI scans that are routinely done at 3-month intervals.

### Statistical analyses

We described baseline characteristics of the cohort as mean and standard deviation (SD) or count and percentage.

Survival was estimated using the Kaplan-Meier method, and between-group comparisons performed through log-rank testing. Comparative analyses of differences in distribution of MGMT promoter methylation, treatment schedule, and type of surgery in PsP or TP were performed using Fisher’s Exact Test. Cox proportional hazard regression model was utilized to assess potential prognostic factors on OS. To estimate the association between clinical and molecular characteristics and PsP in the presence of competing risks (i.e., TP and death), we used the Fine and Gray regression model. Results are reported as hazard ratio (HR) with 95% confidence interval (CI). To evaluate possible confounding of clinical factors, we utilized multivariate analysis. Results are reported as odds ratio (OR) with 95% confidence interval (CI).

Statistical significance was defined as a p-value of less than 0.05. Data analyses were performed using SPSS version 28 and R version 4.4.2.

### Ethics

The study was classified as a non-WMO (Medical Research Involving Human Subjects Act) study, as it did not entail any interventions or actions directly applied to participants for the purpose of medical research. The investigation was limited to the analysis of pre-existing clinical data and complied with institutional and national guidelines governing the use of such data in observational research. Ethical approval was obtained from the review boards of MUMC + and ZMC (METC 16-4-022).

**Fig. 1:**
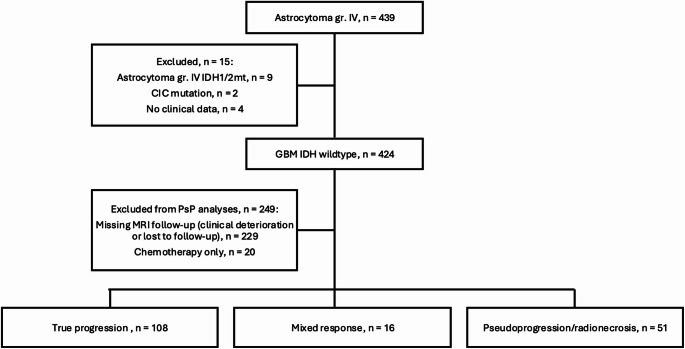
Flowchart of the study population

## Results

### Patient characteristics

A total of 424 patients with GBM were included in the study. Excluding patients that did not have sufficient follow-up MRI scans for PsP, TP and MR determination, as well as patients that only received chemotherapy monotherapy, 175 patients were included for further analyses (Fig. 1). Patient, tumor, and treatment characteristics are shown in Table 1.

**Table 1:** Patient demographics, tumor and treatment characteristics

Characteristics		No (%)
Total		175
Age (years)	< 60	73 (41.7)
	60–69	65 (37.1)
	≥ 70	37 (21.1)
Sex	Male	119 (68.0)
	Female	56 (32.0)
ECOG performance status*	0–1	170 (97.1)
	≥ 2	4 (2.3)
Hemisphere side	Right	94 (53.7)
	Left	77 (44.0)
	Central	2 (1.1)
	Bilateral and/or corpus callosum	2 (1.1)
Lobe location tumor	Frontal	37 (21.1)
	Parietal	21 (12.0)
	Temporal	52 (29.7)
	Occipital	6 (3.4)
	2 lobes involved	45 (25.7)
	3 lobes involved	2 (1.1)
	Midline	1 (0.6)
	Corpus callosum	3 (1.7)
	Insular or frontotemporal	7 (4.0)
	Cerebellar or brainstem	1 (0.6)
MGMT promoter status	Unmethylated	123 (70.3)
	Methylated	51 (29.1)
	Unknown	1 (0.6)
Type of first surgery	Biopsy	57 (32.6)
	(Partial) Resection	118 (64.4)
First line of treatment	Stupp completed	81 (46.3)
	Elderly completed	12 (6.9)
	Stupp halted early	57 (32.6)
	Elderly halted early	7 (4.0)
	Radiotherapy monotherapy	18 (10.3)

SD = standard deviation; MGMT = O6 methylguanine-DNA methyltransferase

*1 patient’s data missing

### Genomic alterations

MGMT methylation was assessed in 99.4% of the patients, with 29.1% of tumors found to exhibit MGMT promoter methylation (Table 2). Further molecular profiling of the tumor (next-generation sequencing and/ or targeted testing) was performed in a subset of 102 patients. The most identified genomic alterations were pathogenic sequencing variants (formerly: mutations) in *TERT*,* TP53*, *PTEN*, *EGFR*, *PIK3CA*, and amplification of EGFR (Table 2). A complete overview of all tested genomic alterations can be found in Supplementary Data table A, as well as the specified alterations in Supplementary Data table B.

**Table 2:** Frequency of pathogenic genomic alterations in the tumor at diagnosis

Genomic Alteration	No. Present (%)	No. Absent (%)	No. Not tested (%)
MGMT promoter methylation	51 (29.1)	123 (70.3)	1 (0.6)
TERT variant	55 (31.4)	24 (13.7)	96 (54.9)
EGFR amplification	38 (21.7)	52 (29.7)	85 (48.6)
TP53 variant	33 (18.9)	45 (25.7)	97 (55.4)
PTEN variant	22 (12.6)	57 (32.6)	96 (54.8)
EGFR variant	20 (11.4)	70 (40.0)	85 (48.6)
PIK3CA variant	10 (5.7)	74 (42.3)	91 (52.0)

### Incidence of PsP, TP and MR

Within our cohort of 175 patients, 108 patients (61.7%) developed TP as their first event upon MRI evaluation, demonstrating increased contrast enhancement with increased perfusion and/or a change in treatment. 67 patients had an MRI with increased contrast enhancement, but without increased perfusion and/or no change in treatment as their first event. Of these 67 patients, 51 (29.1% of total cohort) patients had PsP, as they did not meet TP criteria on subsequent MRI scans within 3 months. The other 16 patients (9.1% of total cohort) had MR, as they did meet TP criteria within 3 months on subsequent MRI scans. Considering the small sample size of the MR group, it was not utilizable for analyses, but purely for creating accurate PsP and TP subgroups for comparisons.

No differences between the PsP and TP groups were observed in MGMT promoter status (*p* = 0.850) and type of surgery (*p* = 0.860). However, there was a significant difference between the groups with regards to chemoradiotherapy completion (*p* = 0.013), with more completion of chemoradiotherapy in the PsP group. The mean time to PsP was 7.9 months (SD 4.2), compared to a mean time to TP of 10.9 months (SD 7.2).

### Overall survival

**Fig. 2:**
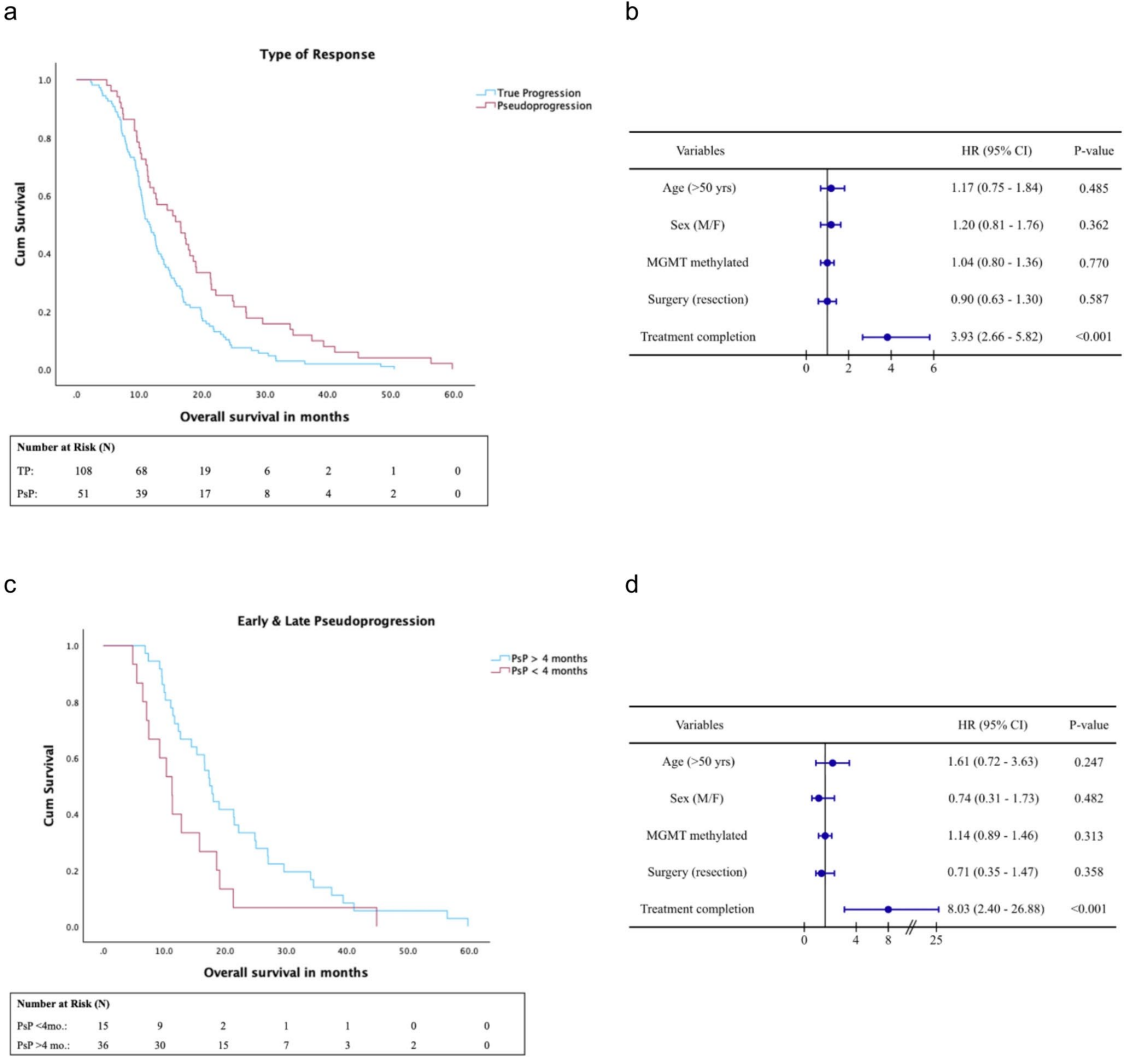
(**a**) OS for first event TP versus MR and PsP. (**b**) Cox proportional hazard model for prognostic factors of OS in **a**. (**c**) OS for early PsP (< 4 months after (chemo)radiotherapy) versus late PsP (> 4 months after (chemo)radiotherapy). (**d**) Cox proportional hazard model for prognostic factors of OS in **c**. HR = hazard ratio; CI = confidence interval

Median OS times for PsP and TP were 16.6 months (95% CI 12.0-21.2), and 11.6 months (95% CI 10.0-13.2), respectively (Fig. 2a). The survival distributions differed significantly (log-rank test, χ² = 8.637, *p* = 0.003).

When splitting the group of patients with PsP by time of occurrence within or after 4 months after concurrent chemoradiotherapy (early versus late PsP), median overall survival time was 11.3 months (95% CI 8.6–13.9, *n* = 15) for the early PsP subgroup and 17.4 months (95% CI 15.4–19.5, *n* = 36) for the late PsP subgroup (log-rank test, χ² = 4.879, *p* = 0.027) (Fig. 2b). When assessing MGMT methylation, the incidence of MGMT promoter methylation did not differ between early or late PsP subgroups (*p* = 0.254).

For both the PsP versus TP and early PsP versus late PsP, Cox proportional hazard models showed that completing concurrent chemoradiation with sequential temozolomide was an independent prognostic factor for OS; age, sex, MGMT methylation and surgery type (biopsy versus resection) were not (Fig. 2b and d). ECOG score was not included as only four patients had a score of 2 or higher.

### Predictive factors for PsP

**Fig. 3:**
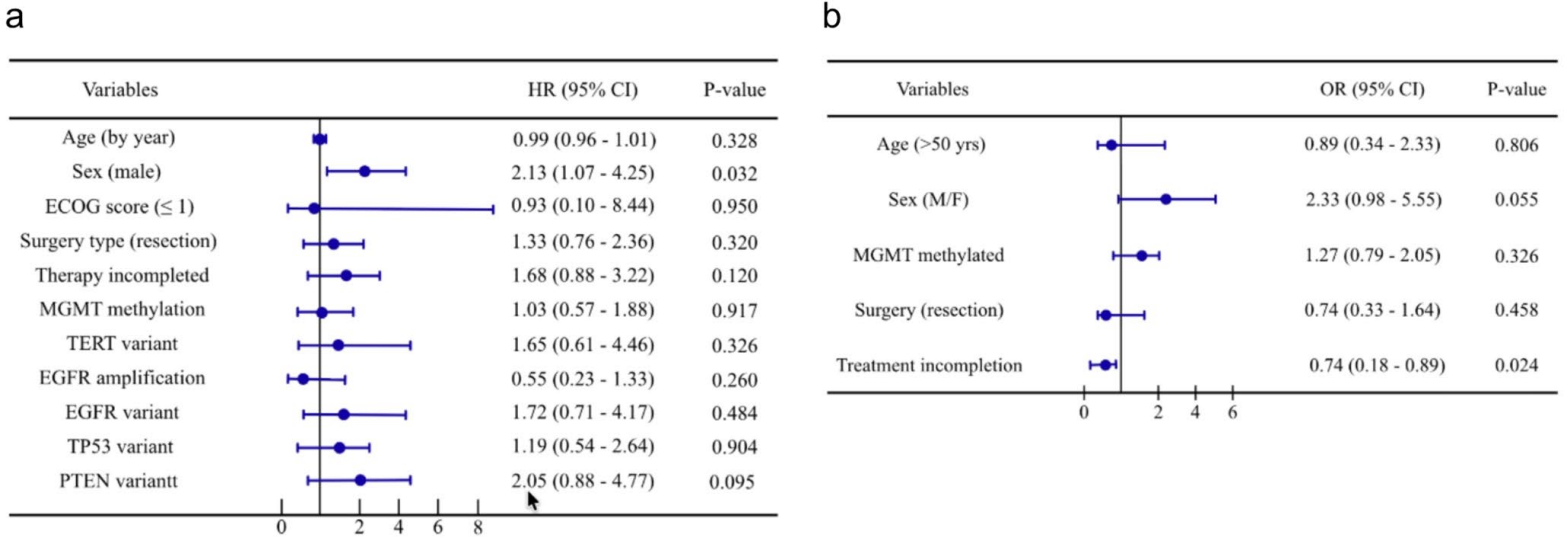
(**a**) Fine and Gray regression analysis of patient, tumor, and treatment characteristics on the occurrence of PsP. (**b**) Multivariate logistic regression model to assess possible confounding of patient and treatment characteristics. HR = hazard ratio; OR = odds ratio; CI = confidence interval; MGMT = O6 methylguanine-DNA methyltransferase; TERT = telomerase reverse transcriptase; EGFR = epidermal growth factor receptor; TP53 = tumor protein p53; PTEN = phosphatase and tensin homolog

The effect of various patient, tumor, and treatment covariates on the occurrence of PsP were analyzed with a Fine and Gray regression model (Fig. 3a). Within this analysis, men had more than twice the chance of PsP compared to women (HR = 2.13, 95% CI: 1.07–4.25, *p* = 0.032), while age, surgery type (biopsy versus (partial) resection), or genomic alterations did not alter the occurrence of PsP. However, our logistic regression analysis for potential confounding demonstrated that male sex is not significantly associated with PsP (OR = 2.33, 95% CI: 0.98–5.55, *p* = 0.055). Not completing concurrent chemoradiation with sequential temozolomide did show to be significantly less associated with PsP (OR = 0.74, 95% CI: 0.18–0.89, *p* = 0.024, while not clearly demonstrating this in the Fine and Gray analysis (Fig. 3b).

To ensure that second-line therapy did not influence our results, we repeated all the analyses with only the patients within our cohort that only received first-line treatment (supplementary data figure A and B). These analyses gave the same results in terms of OS, univariate analysis and multivariate analysis.

## Discussion

This retrospective study evaluated the correlation between various patient, tumor and treatment characteristics of glioblastoma patients and the occurrence of pseudoprogression (PsP). The incidence of PsP observed in our study was 29%, which is in line with the 20–30% reported in previous studies [8–12]. In contrast to other studies, the majority (71%) of PsP was observed beyond the 4-month timepoint after concurrent chemoradiation.

PsP has been associated with improved OS in patients compared to those experiencing early TP [10, 15, 18–20] Our retrospective analysis also shows that PsP reflects a positive response to chemoradiation [10, 34], as we observed a median OS time for patients with PsP of 16.6 months (95% CI 12.0-21.2) for patients that showed PsP first versus 11.6 months (95% CI 10.0-13.2) for patients who did not have PsP before TP. In addition, we observed a 6-month shorter median OS in patients with early PsP (within 4 months post-radiation) than patients with late PsP. A probable explanation is that patients with early PsP more often could not complete the Stupp or elderly treatment scheme due to rapid clinical deterioration (29% therapy completion in the early group as compared to 89% in the late group). Completion of Stupp/elderly treatment schedule is a known prognostic factor for OS [35]. Indeed, patients who did not complete Stupp/Elderly treatment had an 8-month shorter median OS compared to patients who did complete concurrent chemoradiation with temozolomide (Supplementary data figure C).

Several factors potentially associated with PsP have been studied. In our study, we analyzed various patient, tumor, and treatment characteristics. We found that males have a 2.13 times higher hazard rate for PsP compared to females in our univariate analysis. However, in the multivariate analysis the result was not significant, demonstrating confounding from other clinical factors. ECOG score (HR = 0.93, 95% CI: 0.10–8.44, *p* = 0.950) was not significantly associated with an increased likelihood of PsP. A significant higher Karnofsky Performance Status score has previously been observed in patients with PsP compared to those with TP [36], but we did not replicate this finding. This can be attributed to the low number of poor condition patients included within our study, as these patients typically receive partial or no therapy. Patients who underwent a biopsy did not have a significant risk difference of PsP that patients who underwent (partial) resection, in line with the results of a previous study [37]. Completing concurrent chemoradiation (either Stupp or elderly) with sequential chemotherapy has also not shown to be significantly associated with PsP in our univariate analysis but was significantly associated in the multivariate analysis. Although it is known that completing Stupp/Elderly treatment has a positive effect on OS, previous studies have not directly evaluated complete or incomplete treatment with respect to the development of PsP. Evaluating this with larger cohorts would therefore be of interest, also to confirm our significant result from the multivariate analysis.

In terms of molecular analyses, MGMT promoter methylation has been the most extensively studied. Multiple studies have shown that MGMT promoter methylation is associated with an increased likelihood of PsP [8, 10, 23–26]. Nevertheless, in our study, we did not observe a significant correlation between MGMT promoter methylation and the occurrence of PsP in comparison to MR and TP, despite similar sample sizes as in previous studies. An explanation could be the heterogenic use of MGMT assays between this study and previous studies, as well as varying methylation thresholds that have been utilized. Supplementary Data Table C provides a comprehensive overview of studies that have evaluated MGMT promoter methylation with regards to PsP development. Notably, many studies do not describe the methylation threshold, and when a threshold is mentioned, it is 9–10%. When comparing these thresholds to our higher thresholds (MSP 15% and MLPA 25%), significantly more patients are considered unmethylated in our cohort, leading to different methylation and PsP correlation results, possibly causing the statistically insignificant difference between PsP and TP.

A limited number of glioma studies have explored pathological genomic alterations, including sequencing variants (formerly, mutations) in *TP53*, *PTEN*, *TERT*, and copy number variation of *EGFR* (amplification) in relation to PsP. We did not observe an association between these common genomic alterations and the occurrence of PsP. In literature, findings on the relation between *TP53* sequencing variants and PsP are inconsistent, with one study suggesting a correlation with PsP, while another found no such correlation [9, 18]. While genomic alterations of *PTEN*, *EGFR*, and *TERT* have been examined in some studies, this research has been limited, and no significant associations with PsP have been reported to date [24, 25, 38].

The limitations of this study are inherent to its retrospective design. Selection bias may have influenced the findings, as the study population was predefined based on available historical records, which may not fully represent the broader patient population. Moreover, a large fraction of the cohort (229 patients) had to be excluded due to missing data, possibly affecting the distribution of variables and outcomes in the final study sample. The reliance on existing medical records introduces the possibility of incomplete or inconsistent data, particularly regarding tumor and genomic characteristics. Having used MRI reports by neuroradiologists instead of reviewing MRI scans directly is also a limiting reliability factor in our study. Additionally, confounding factors that were not accounted for or recorded may have impacted the observed associations, limiting the ability to establish causal relationships. Another difficulty within this study topic is the definition of PsP, and how to properly distinguish it from TP as well as radiation necrosis [39]. As mentioned previously, the RANO criteria define PsP most accurately with stabilizing radiological changes and no change in treatment during follow-up. This still means that PsP can only be established retrospectively, which delays adequate therapy changes. In our cohort, we optimized PsP and TP subgroup defining by creating a separate MR group. The MR group could not be utilized in analyses due to the small sample size. Regardless, comparing PsP and TP is clinically more relevant. Furthermore, we did utilize rCBV to further distinguish TP from PsP. Although rCBV is not specific for PsP, we do believe it can aid in the distinguishing with TP, as based on more recent publications related to the RANO criteria as well as independent studies on perfusion MRI [14, 22, 33]. Unfortunately, in our cohort the consistency of reporting rCBV was limited due to older time points when perfusion tests were not standardly used yet, having mixed reporting of perfusion. Therefore, we did also score increased contrast enhancement and a change in treatment as TP, possibly influencing the accurate incidences of PsP and TP in our cohort. Overall, having a uniform but improved PsP definition would allow for more prospective PsP analyses. This can be achieved by re-evaluating previous definitions, and further validating novel radiological modalities, including magnetic resonance spectroscopy imaging (MRSI) and Fluoroethyl-L-Tyrosine Positron Emission Tomography (FET-PET) MRI imaging [36, 39]. Lastly, our analysis was limited by missing somatic genomic data, as genetic aberration testing in clinical practice for GBM has only been extensively utilized in recent years.

In conclusion, in this retrospective cohort PsP, especially PsP observed at more than 4 months after chemoradiotherapy, was associated with improved OS compared to TP as the initial event. Completing chemoradiotherapy was the only significant factor positively associated with OS in the PsP group compared to the TP group. With regards to PsP occurrence, male sex and completing chemoradiation were the evaluated factors with significant association in either the univariate or multivariate analysis. MGMT promoter methylation was not associated with the occurrence of PsP in our patient cohort. Our findings suggest that establishing uniform MGMT methylation thresholds of the different methylation platforms that are currently in use world-wide is urgently needed to establish the predictive value of MGMT promoter methylation and the development of PsP. In addition, patient cohorts with full coverage of somatic genomics are urgently needed to investigate other potential genomic drivers of PsP. To facilitate an accurate depiction of these factors on PsP, the definition of PsP should be uniform and improved, by using new radiological modalities such as MRSI and FET-PET.

## Supplementary Information

Below is the link to the electronic supplementary material.

Supplementary Material 1

## Data Availability

Data availability by corresponding author on reasonable request.
