# Supplementary material for: Clinical predictors of pseudoprogression in glioblastoma: a retrospective cohort analysis
Source: J Neurooncol. 2025 Oct 24;176(1):42. doi: 10.1007/s11060-025-05299-0 (PMC12552241; doi:10.1007/s11060-025-05299-0)
Supplement: Supplementary file 1 — Supplementary Material 1 [file 11060_2025_5299_MOESM1_ESM.docx]

Supplementary Data

*Table A: Comprehensive Listing of (Likely) Pathogenic Genomic Alterations Tested in Cohort.*

| **Alteration** | **Present (N)** | **Absent (N)** | **Not tested (N)** |
| --- | --- | --- | --- |
| MGMT promoter methylation | 51 (29.1%) | 123 (70.3%) | 1 (0.6%) |
| TERT variant | 55 (31.4%) | 24 (13.7%) | 96 (54.9%) |
| EGFR amplification | 38 (21.7%) | 52 (29.7%) | 85 (48.6%) |
| TP53 variant | 33 (18.9%) | 45 (25.7%) | 97 (55.4%) |
| PTEN variant | 22 (12.5%) | 57 (32.6%) | 96 (54.9%) |
| EGFR variant | 20 (11.4%) | 70 (40.0%) | 85 (48.6%) |
| PIK3CA variant | 10 (5.7%) | 74 (42.3%) | 91 (52.0%) |
| RB1 variant | 6 (3.4%) | 65 (37.1%) | 104 (59.5%) |
| MDM2 variant | 6 (3.4%) | 65 (37.1%) | 104 (59.5%) |
| PDGFRA variant | 2 (1.1%) | 79 (45.2%) | 94 (53.7%) |
| ABL1 variant | 2 (1.1%) | 72 (41.2%) | 101 (57.7%) |
| PTPN11 variant | 2 (1.1%) | 68 (38.9%) | 105 (60.0%) |
| ATM variant | 2 (1.1%) | 68 (38.9%) | 105 (60.0%) |
| PDGFRA/KIT/KDR co-amplification | 2 (1.1%) | 66 (37.8%) | 107 (61.1%) |
| CDK4 variant | 2 (1.1%) | 1 (0.6%) | 172 (98.3%) |
| H3F3A variant | 2 (1.1%) | 0 (0.0%) | 173 (98.9%) |
| BRAF variant | 1 (0.6%) | 83 (47.4%) | 91 (52.0%) |
| MET variant | 1 (0.6%) | 83 (47.4%) | 91 (52.0%) |
| ALK variant | 1 (0.6%) | 82 (46.9%) | 92 (52.5%) |
| KIT variant | 1 (0.6%) | 81 (46.3%) | 93 (53.1%) |
| FGFR3 variant | 1 (0.6%) | 79 (45.1%) | 95 (54.3%) |
| FGFR1 variant | 1 (0.6%) | 77 (44.0%) | 97 (55.4%) |
| SMAD34 variant | 1 (0.6%) | 68 (38.9%) | 106 (60.5%) |
| CDH1 variant | 1 (0.6%) | 68 (38.9%) | 106 (60.5%) |
| CTNBB1 variant | 1 (0.6%) | 68 (38.9%) | 106 (60.5%) |
| ATRX variant | 1 (0.6%) | 0 (0.0%) | 174 (99.4%) |
| CDK6 variant | 1 (0.6%) | 0 (0.0%) | 174 (99.4%) |
| RET variant | 0 (0.0%) | 75 (42.9%) | 100 (57.1%) |
| APC variant | 0 (0.0%) | 69 (39.4%) | 106 (60.6%) |
| CIC variant | 0 (0.0%) | 69 (39.4%) | 106 (60.6%) |
| NRAS variant | 0 (0.0%) | 15 (8.6%) | 160 (91.4%) |
| KRAS variant | 0 (0.0%) | 14 (8.0%) | 161 (92.0%) |
| AKT1 variant | 0 (0.0%) | 13 (7.4%) | 162 (92.6%) |
| HRAS variant | 0 (0.0%) | 13 (7.4%) | 162 (92.6%) |
| JAK2 variant | 0 (0.0%) | 13 (7.4%) | 162 (92.6%) |
| AKT2 variant | 0 (0.0%) | 12 (6.9%) | 163 (93.1%) |
| ERBB2 variant | 0 (0.0%) | 14 (8.0%) | 161 (92.0%) |
| AKT3 variant | 0 (0.0%) | 7 (4.0%) | 168 (96.0%) |
| ARAF variant | 0 (0.0%) | 7 (4.0%) | 168 (96.0%) |
| DDR2 variant | 0 (0.0%) | 7 (4.0%) | 168 (96.0%) |
| GNA11 variant | 0 (0.0%) | 7 (4.0%) | 168 (96.0%) |
| GNAQ variant | 0 (0.0%) | 7 (4.0%) | 168 (96.0%) |
| GNAS variant | 0 (0.0%) | 7 (4.0%) | 168 (96.0%) |
| MAP2K1 variant | 0 (0.0%) | 7 (4.0%) | 168 (96.0%) |
| MTOR variant | 0 (0.0%) | 7 (4.0%) | 168 (96.0%) |
| POLE variant | 0 (0.0%) | 7 (4.0%) | 168 (96.0%) |
| RAF1 variant | 0 (0.0%) | 7 (4.0%) | 168 (96.0%) |
| ROS1 variant | 0 (0.0%) | 7 (4.0%) | 168 (96.0%) |
| MSH2 variant | 0 (0.0%) | 6 (3.4%) | 169 (96.6%) |
| MSH6 variant | 0 (0.0%) | 6 (3.4%) | 169 (96.6%) |
| PMS2 variant | 0 (0.0%) | 6 (3.4%) | 169 (96.6%) |
| FLT3 variant | 0 (0.0%) | 5 (2.9%) | 170 (97.1%) |
| MLH1 variant | 0 (0.0%) | 5 (2.9%) | 170 (97.1%) |
| FGFR2 variant | 0 (0.0%) | 3 (1.7%) | 172 (98.3%) |
| CDKN2A variant | 0 (0.0%) | 2 (1.1%) | 173 (98.9%) |
| MLH6 variant | 0 (0.0%) | 2 (1.1%) | 173 (98.9%) |
| MSH5 variant | 0 (0.0%) | 1 (0.6%) | 174 (99.4%) |
| FUBP1 variant | 0 (0.0%) | 0 (0.0%) | 175 (100%) |
| NOTCH1 variant | 0 (0.0%) | 0 (0.0%) | 175 (100%) |

*Table B: Specified Variants of Genetic Aberrations in Table B.*

| **Gene** | **Specific mutation** |
| --- | --- |
| TERT variant | p. C250T, p. C228T, p. C209T, p. C113T, p. C135T |
| TP53 variant | p. G245S, p. R175H, p. V157D, p. V216M, p. I255T, p. R248W, p. E286K, p. C275Y, p. R273H, p. R158H, p. M246I, p. C176F, p. P72R, p. R196*, p. R280K, p. P190L, p. V97I, p. C238Y, p. Y103*, p. I254N, p. R110L, p. L265Q, p. C135*, p. A175C, p. A213*, p. A249S, p. G244C, p. T125A, p. P278S, p. I195T, p. R213* |
| PTEN variant | p. K125N, p. G251D, p. K6E, p. C124R, p. Y16*, p. R15I, p. R233*, p. D326N, p. L122P, p. M134I, p. R15S, p. G165E, p. E284*, p. G127E, p. R47S, p. T319L*2, p. T319T, p. V217P, p. Q149* |
| EGFR variant | p. D256G, p. A289T, p. A289V, p. R108K, p. H304Y, p. R324L, p. T263P, p. G598V, p. T725M, p. P848L |
| PIK3CA variant | p. E542K, p. G1049T, p. H1047R, p. N345K, p. Q546L, p. Q546R, p. H1047A, p. G118A |
| RB1 variant | p. T728*, p. G280*, p. L769*, p. Q685* |
| MDM2 variant | Unknown^1^ |
| PDGFRA variant | p. V658A, p. P567L |
| ABL1 variant | p. K247R, p. K266R |
| PTPN11 variant | p. G76L |
| ATM variant | p. A1309T |
| CDK4 variant | Unknown^1^ |
| H3F3A variant | p. L28M |
| BRAF variant | p. V600G |
| MET variant | Unknown^1^ |
| ALK variant | p. R1231W |
| KIT variant | p. C840Y + p. C844Y |
| FGFR3 variant | c.445+2_445+5del4 |
| FGFR1 variant | p. D124N + p. D43N + p. D165N + p. D132N |
| SMAD34 variant | p. G243E |
| CDH1 variant | p. T340M |
| CTNBB1 variant | p. Q28* |
| MSH2 variant | Unknown^1^ |
| MSH6 variant | Unknown^1^ |
| PMS2 variant | Unknown^1^ |
| MLH1 variant | Unknown^1^ |
| ATRX variant | p. A1940V |
| CDK6 variant | Unknown^1^ |

^1^Lost variant data as variants were tested in other academic hospitals, from where data was no longer available.

*Table C: Overview Previous Study Results MGMT-PsP Correlation*

| **Study** | **Cohort** | **MGMT Assay** | **Outcomes** | **Conclusion** |
| --- | --- | --- | --- | --- |
| Brandes et al. *Journal of Clinical Oncology* (2008). | Retrospective analysis of 103 GBM tissue biopsies (30% PsP). | Methylation specific polymerase chain reaction (MSP). No methylation threshold or CpG site information. | 91.3% probability of PsP in methylated MGMT, 41% probability in unmethylated MGMT. | Higher incidence of PsP with methylated MGMT. |
| Motegi et al. *Brain Tumor Pathology* (2013). | Retrospective study of 32 patients (9.4% PsP). | Methylation specific polymerase chain reaction (MSP). No methylation threshold or CpG site information. | Fisher Exact test association MGMT and PsP, P=.424. | No significant association between MGMT and PsP.  Very small sample size. |
| Balana et al. *Cancer Medicine* (2017). | Retrospective analysis of 256 patients with chemoradiation (21.9% PsP). | Methylation-specific PCR. No methylation threshold or CpG site information. | Occurrence of PsP as opposed to PD; OR 3.48 (95% CI 1.606-7.564, P=0.002). | Significant higher chance of PsP with MGMT methylation. |
| Li et al. *Clinical Neurology and Neurosurgery* (2016). | Retrospective analysis of 145 patients (26% PsP). | Methylation specific polymerase chain reaction (MSP). No methylation threshold or CpG site information. | 1.336 times probability of PsP with MGMT methylation.  OR 3.805 (95% CI 1.687-8.578, P=0.001). | MGMT independent risk factor for PsP. |
| Li et al. *Frontiers in Oncology* (2021). | Retrospective analysis of 234 patients (21.6% PsP). | Pyrosequencing (PSQ), CpG sites 74-81. MGMT methylation threshold ≥9%. No specific CpG information. | OR 9.74 (95% CI 4.46-21.24, P<0.001). | MGMT associated factor with PsP. |
| Hagiwara et al. *Journal of Neuro-Oncology* (2022). | Retrospective analysis of 169 patients (38.5% PsP). | Not described in article. No methylation threshold or CpG site information. | 12.5% MGMT methylation in PD group, 26.2% in PsP group, P=0.02 (Chi-squared Test) | Association of MGMT with PsP. |
| Blakstad et al. *Neuro-Oncology Practice* (2023). | Cohort study of 284 patients (19.4% PsP after 3 months). | Pyrosequencing (PSQ), methylation threshold 10%. No methylation threshold or CpG site information. | OR 9.13 (95% CI 3.78-22.02), P<0.001). | MGMT significantly associated factor with PsP. |

| a  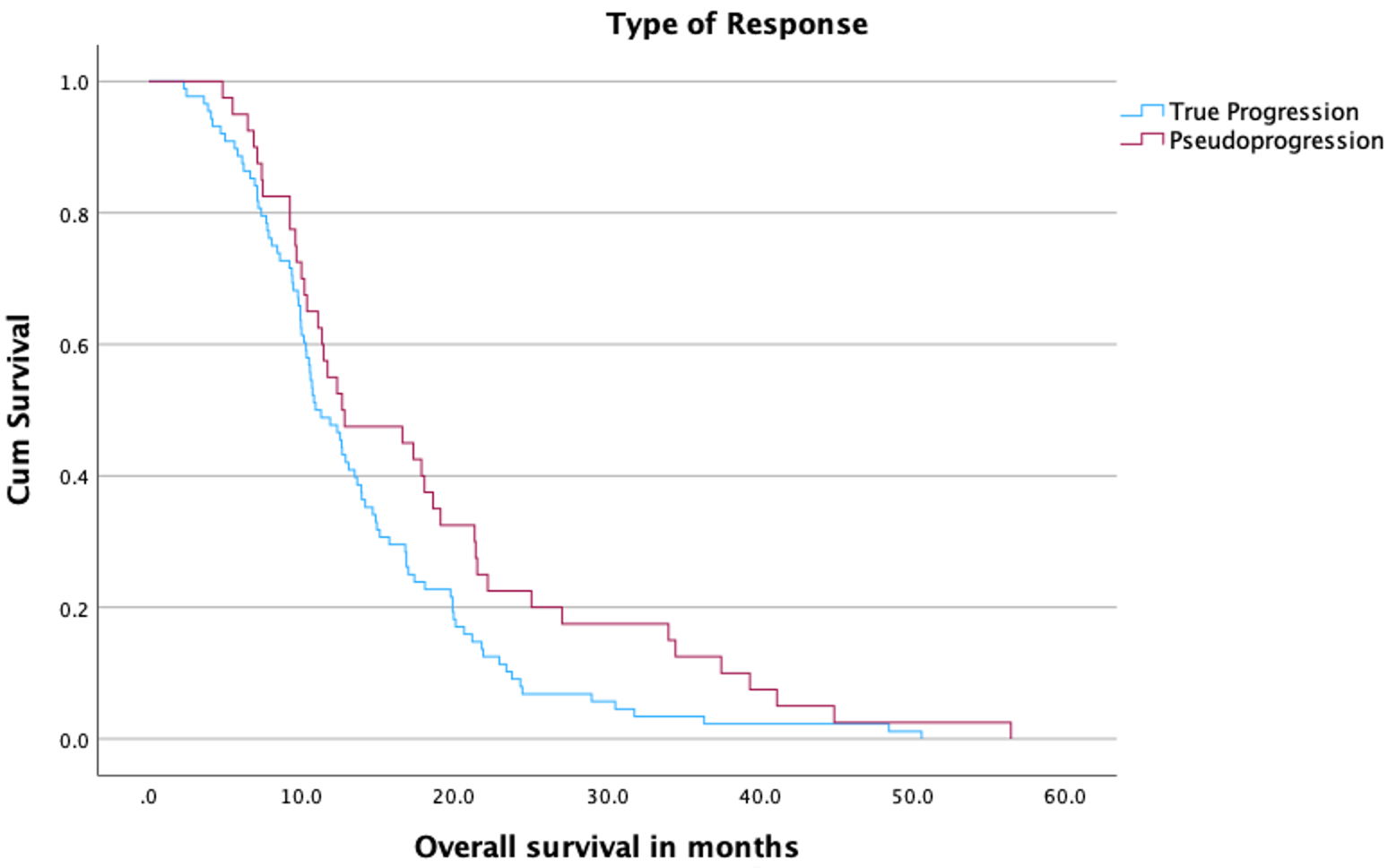 | b  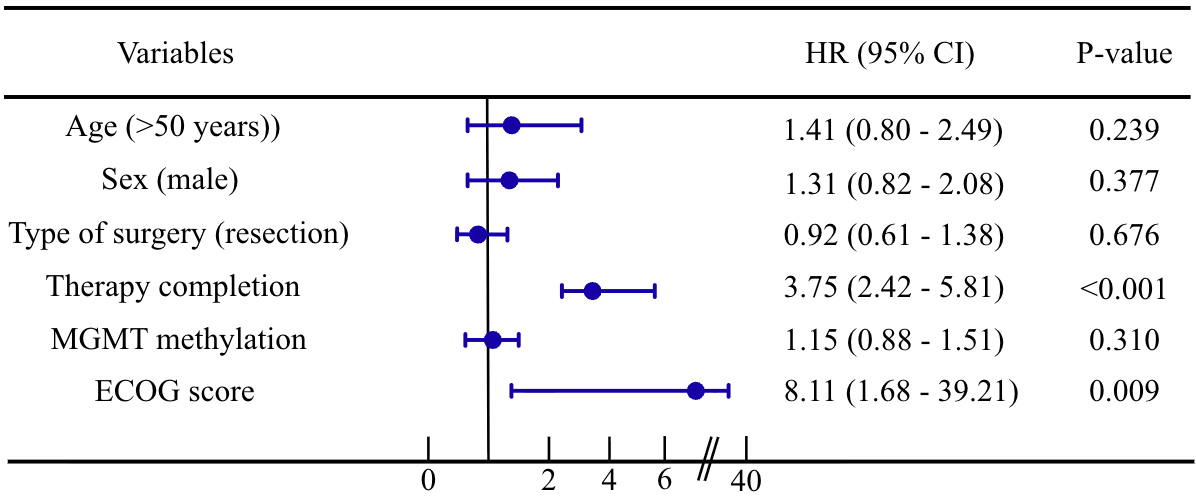 |
| --- | --- |
| c  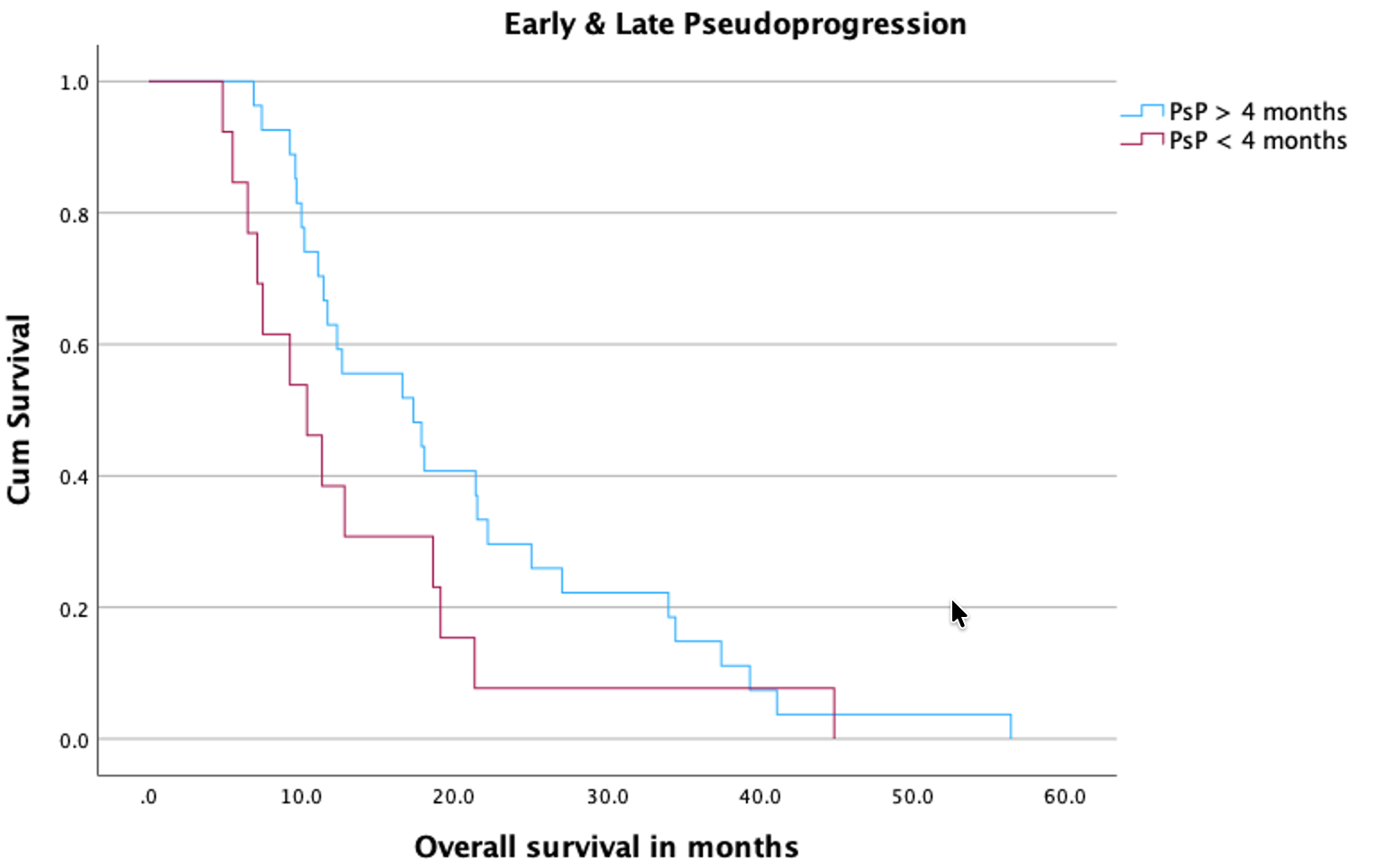 | d  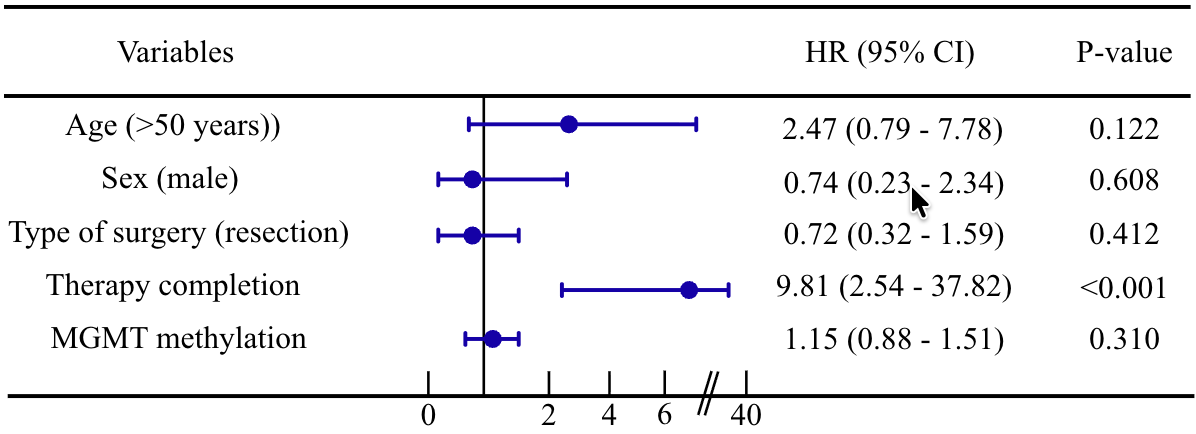 |

***Figure A****: survival analyses with including only patients without second-line therapy.* ***(a)*** *OS for first event TP versus MR and PsP.* ***(b)*** *Cox proportional hazard model for prognostic factors of OS in figure 2a.* ***(c)*** *OS for early PsP (<4 months after (chemo)radiotherapy) versus late PsP (>4 months after (chemo)radiotherapy).* ***(d)*** *Cox proportional hazard model for prognostic factors of OS in figure 2c.*

*HR = hazard ratio; CI = confidence interval*

| a  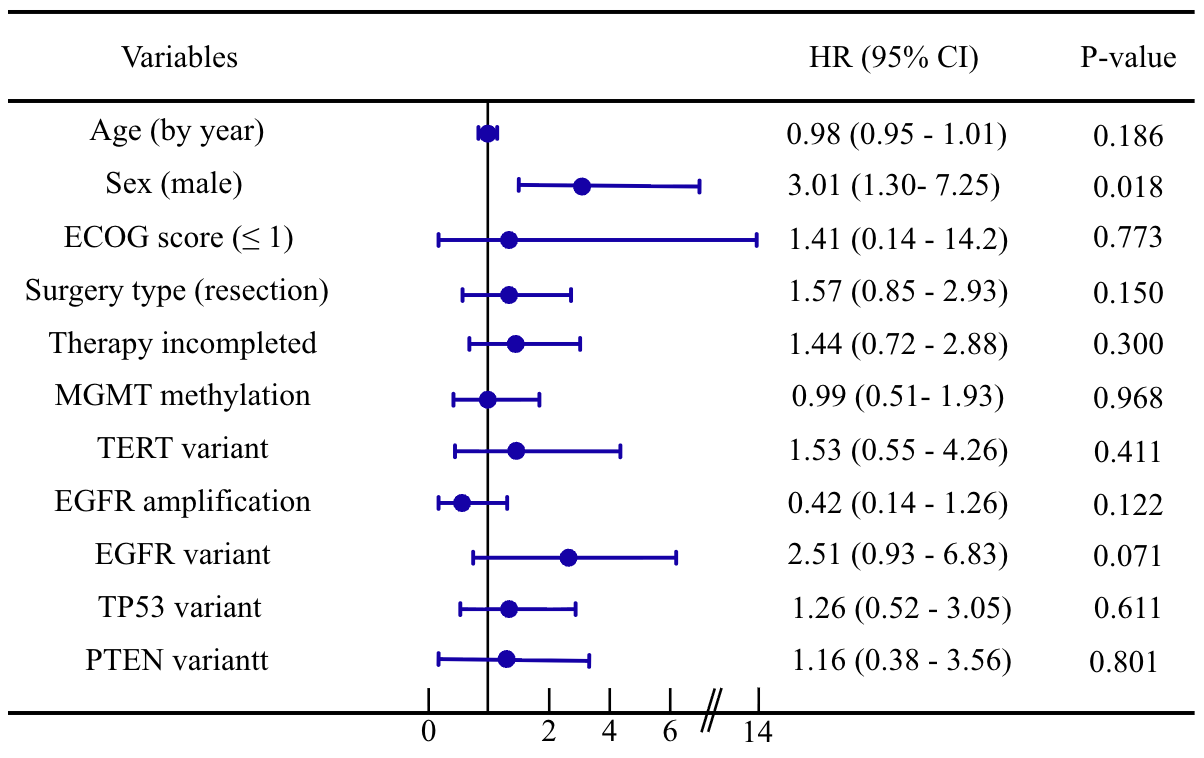 | b  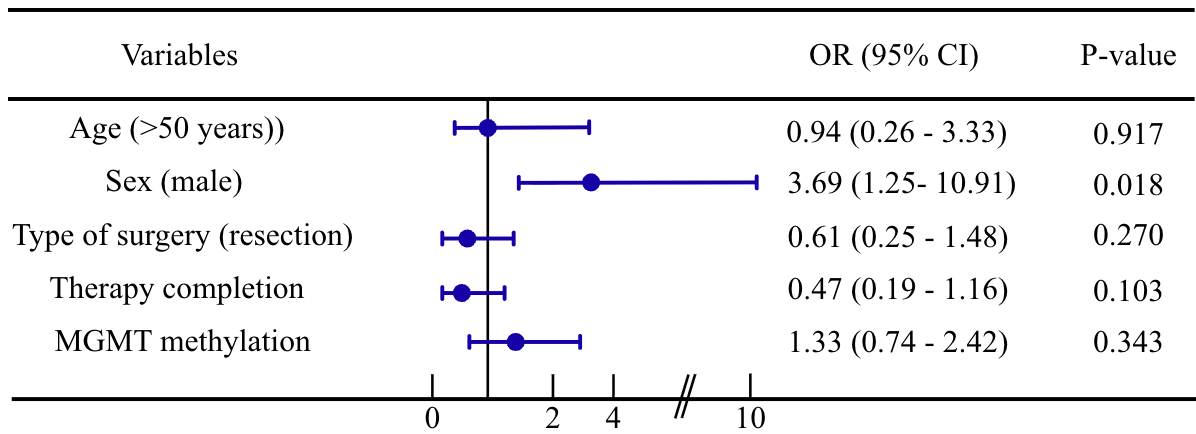 |
| --- | --- |

***Figure B****: univariate and multivariate analyses repeated on patients without second-line therapy.* ***(a)*** *Fine and Gray regression analysis of patient, tumor, and treatment characteristics on the occurrence of PsP.* ***(b)*** *Multivariate logistic regression model to assess possible confounding of patient and treatment characteristics. HR = hazard ratio; OR = odds ratio; CI = confidence interval; MGMT = O6 methylguanine-DNA methyltransferase; TERT = telomerase reverse transcriptase; EGFR = epidermal growth factor receptor; TP53 = tumor protein p53; PTEN = phosphatase and tensin homolog*


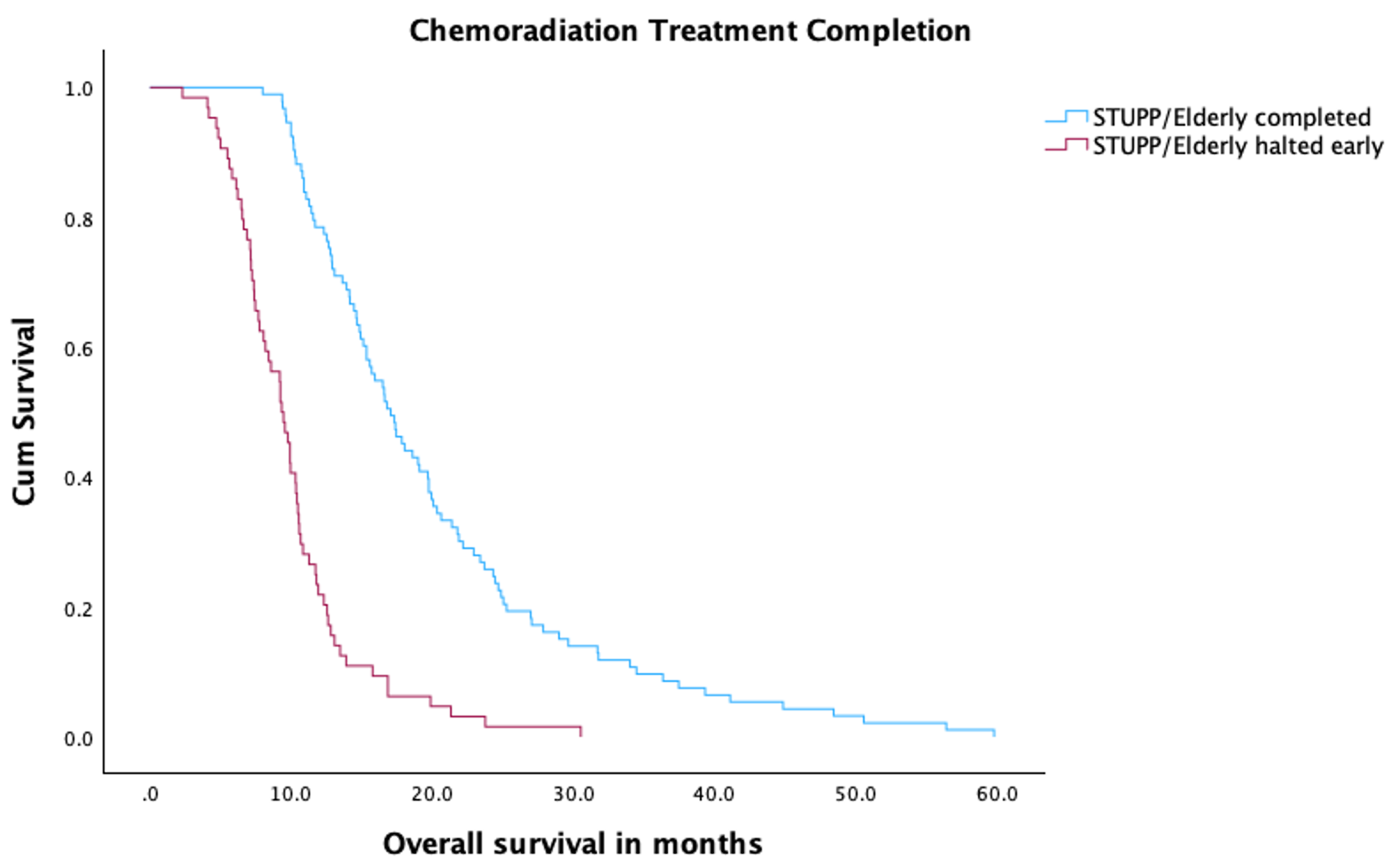


***Figure C****: Overall survival for completed and incomplete concurrent chemoradiation (either Stupp or elderly).* Log-rank test, χ² *71.483, p < 0.001.*
